# Concurrent query processing in a GPU-based database system

**DOI:** 10.1371/journal.pone.0214720

**Published:** 2019-04-16

**Authors:** Hao Li, Yi-Cheng Tu, Bo Zeng

**Affiliations:** 1 Department of Computer Science and Engineering, University of South Florida, Tampa, FL, United States of America; 2 Department of Industrial Engineering, University of Pittsburgh, Pittsburgh, PA, United States of America; King Abdulaziz University, SAUDI ARABIA

## Abstract

The unrivaled computing capabilities of modern GPUs meet the demand of processing massive amounts of data seen in many application domains. While traditional HPC systems support applications as standalone entities that occupy entire GPUs, there are GPU-based DBMSs where multiple tasks are meant to be run at the same time in the same device. To that end, system-level resource management mechanisms are needed to fully unleash the computing power of GPUs in large data processing, and there were some researches focusing on it. In our previous work, we explored the single compute-bound kernel modeling on GPUs under NVidia’s CUDA framework and provided an in-depth anatomy of the NVidia’s concurrent kernel execution mechanism (CUDA stream). This paper focuses on resource allocation of multiple GPU applications towards optimization of system throughput in the context of systems. Comparing to earlier studies of enabling concurrent tasks support on GPU such as MultiQx-GPU, we use a different approach that is to control the launching parameters of multiple GPU kernels as provided by compile-time performance modeling as a kernel-level optimization and also a more general pre-processing model with batch-level control to enhance performance. Specifically, we construct a variation of multi-dimensional knapsack model to maximize concurrency in a multi-kernel environment. We present an in-depth analysis of our model and develop an algorithm based on dynamic programming technique to solve the model. We prove the algorithm can find optimal solutions (in terms of thread concurrency) to the problem and bears pseudopolynomial complexity on both time and space. Such results are verified by extensive experiments running on our microbenchmark that consists of real-world GPU queries. Furthermore, solutions identified by our method also significantly reduce the total running time of the workload, as compared to sequential and MultiQx-GPU executions.

## Introduction

**Fig 1:**
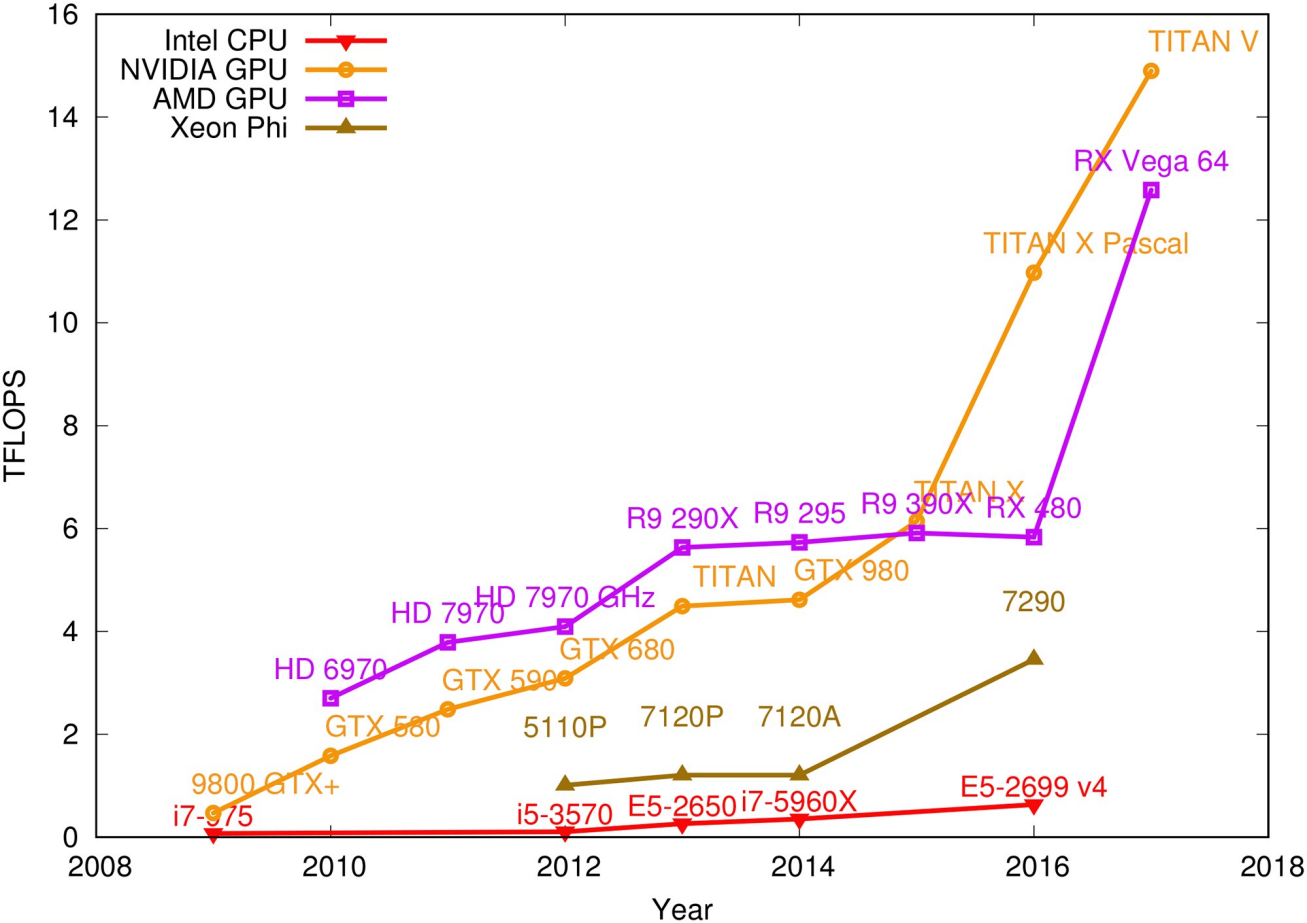
Growth of computing capacity on Intel CPU, Intel Phi co-processor, and NVidia/AMD GPUs. Data extracted from [1, 2].

With the recent development of semiconductor technology, the number of processing units integrated on a chip increases rapidly, resulting in massively parallel computing capability. Many-core hardware systems such as Intel Xeon Phi co-processors and Graphics Processing Units (GPU) are becoming more and more popular. As shown in Fig 1, the single precision peak performance of the latest NVidia GPU reaches 14.899 TFLOPS and the latest AMD GPU has 12.583 TFLOPS. On contrary, the CPU only provides 0.634 TFLOPS, and the Intel Phi reaches 3.456 TFLOPS. Such unrivaled computing power has made GPUs an indispensable component in today’s high-performance computing (HPC) systems and shown great value in many compute-intensive applications.

The use of GPUs in application domains that typically are not heavy users of HPC resources is also explored. For example, novel database management system (DBMS) architectures based on GPGPU have been proposed [3–6] to meet the challenges of querying large-scale data. Commercial systems such as *MapD* [7] and *Kinetica* [8] have seen success in the business world. Note that, although traditional databases are I/O-bound systems, the above works all focus on scenarios that are computation-bound.

They are either explicitly defined as in-memory databases [3, 8] or adopted a “push-based” design in its system architecture [6]. Unlike traditional relational DBMSs, the core of a push-based DBMS [9] follows a stream-based design in its data input mechanism. In particular, it creates shared I/O streams to deliver data to all running queries simultaneously, while traditional DBMSs (“pull-based” system) retrieve the needed data from storage for each individual query. Due to the single I/O stream and minimization of random I/Os, push-based DBMSs can efficiently process very large data with a low I/O cost. With the large data and the complex queries such databases are meant to process nowadays, the performance bottleneck is essentially moved from I/O to computation, and parallel hardware such as GPUs fills this gap. It is worth mentioning that, DBMS support of data analytics has also been explored [10–12]—this requires more in-core computing power than traditional data retrieval queries. Besides database community, many other domains such as scientific computing [13, 14] adopt similar ideas in building system software with data streaming design and use of GPUs to achieve remarkable performance. Proposals to handle infinite data streams in GPUs are also explored [4]. In our previous work [6], we proposed a GPGPU-based Scientific Data Management System (G-SDMS) that uses CUDA-supported GPUs as the platform for query processing in a push-based manner. G-SDMS can be viewed as a middleware that provides query processing/optimization and resource management functionalities on top of CUDA.

They are either explicitly defined as in-memory databases [3, 8] or adopt a “push-based” design in its system architecture [6]. Unlike traditional relational DBMSs, the core of a push-based DBMS [9] follows a stream-based design in its data input mechanism. In particular, it creates shared I/O streams to deliver data to all running queries simultaneously, while traditional DBMSs (“pull-based” system) retrieve the needed data from storage for each individual query. Due to the single I/O stream and minimization of random I/Os, push-based DBMSs can efficiently process very large data with a low I/O cost. With the large data and the complex queries such databases are meant to process nowadays, the performance bottleneck is essentially moved from I/O to computation, and parallel hardware such as GPUs fills this gap. It is worth mentioning that, DBMS support of data analytics has also been explored [10–12]—this requires more in-core computing power than traditional data retrieval queries. Besides database community, many other domains such as scientific computing [13, 14] adopt similar ideas in building system software with data streaming design and use of GPUs to achieve remarkable performance. Proposals to handle infinite data streams in GPUs are also explored [4]. In our previous work [6], we proposed a GPGPU-based Scientific Data Management System (G-SDMS) that uses CUDA-supported GPUs as the platform for query processing in a push-based manner. G-SDMS can be viewed as a middleware that provides query processing/optimization and resource management functionalities on top of CUDA.

A key challenge in building aforementioned systems is to support concurrent execution of heterogeneous tasks (i.e., queries). In push-based DBMSs, queries are inherently concurrent—data is loaded into the memory chunk by chunk and all queries have to be processed against the *in situ* chunk before the next chunk is loaded. Even in in-memory databases, concurrent processing of a group of queries that are issued under different timestamps are shown to outperform traditional single-query processing [5]. In other words, such systems are optimized towards data processing throughput (rather than response time of individual tasks) therefore maximizing resource utilization is essential. In a CPU-based environment, (main) memory and CPU cycles are often the only involved resources, and much work has been done in the context of data stream systems [15]. The GPUs, on the other hand, have a complex architecture that provides abundant resources under more categories (e.g., registers, shared memory, blocks, threads, etc.). Such complexity brings opportunities for improved application performance, and also necessitates non-trivial modeling and algorithmic techniques in system design and implementation.

In recent years, a number of studies also have explored the potential parallelism of speeding up database operations on GPUs [5, 16–18]. Most of them [5, 16, 18] involves rewriting existing systems and only optimizing towards single query, but in [17], Wang *et al*. implemented MultiQx-GPU, a system consists of query scheduler and device memory manager, that can be built in the query engine and resides in the application level like a middleware. It is capable of executing queries as well as doing in-database analytics. MultiQx-GPU provides concurrency among different query engines systems or even non-query tasks by sharing GPU resources. However, the concurrency achieved by MultiQx-GPU is mostly from controlling the workload from CPU side and overlapping the I/O between CPU and GPU, it does not implement the resource sharing in GPUs due to lack of knowledge of GPU resource allocation mechanism at that time. We use MultiQx-GPU to benchmark our work in this paper, and a detailed introduction to it can be found in ***Overview of MultiQx-GPU***.

### Overview of our approach

In this paper, we present a general scheme in optimizing the concurrency and overall performance of heterogeneous (parallel) tasks under the Compute Unified Device Architecture (CUDA) environment [19].

Similar to traditional DBMSs, query processing algorithms on GPUs are designed at the relational operator level. Each algorithm could be divided into multiple parallel functions in GPUs called *kernels*. For example, for Query slowromancapi@ that scans a single table *R*, the involved kernels are: *scanning the tuples that meet the condition*, and *outputting results*; for Query slowromancapii@ that performs a hash join between tables *R* and *S*, we have the following kernels: *building hash table of R*, *scan R*, *building hash table of S*, *scanning the matching tuples*, and *outputting the results*.

**Fig 2:**
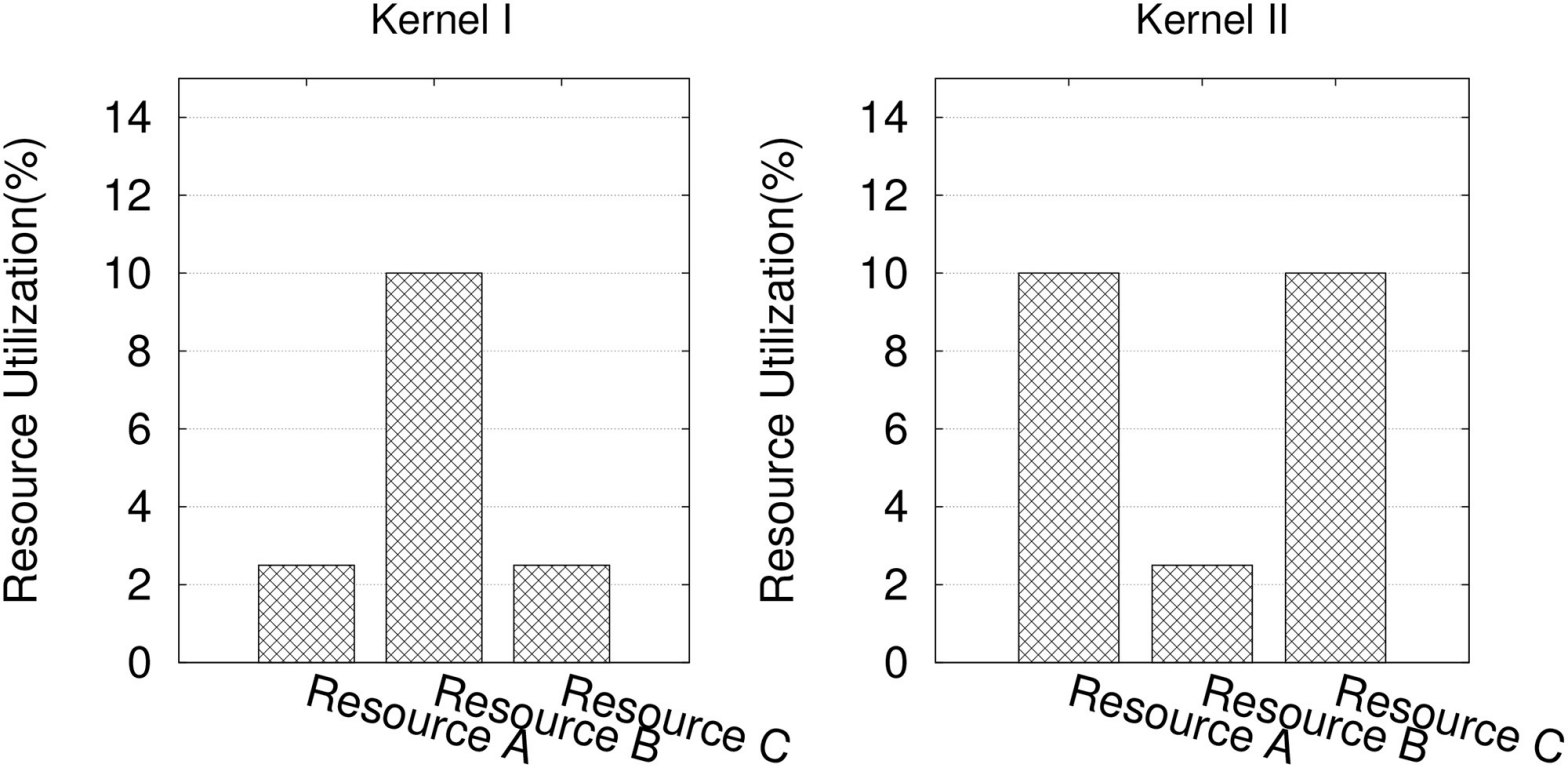
Normalized resource use per thread of two different kernels.

CUDA allows a kernel to run with a large number of threads and blocks. The limited total resource, however, means the threads will have to take turns to be executed on the hardware. To run a thread in a CUDA kernel, a certain amount of resource under different categories is required. In a multi-kernel environment, it is essential to *determine how many threads for each kernel should be launched simultaneously such that the overall performance is the best*. Being the main objective of our study, this problem is non-trivial due to the multiple types of resources involved. Let us illustrate this with a simple example with two kernels bearing different resource use patterns (Fig 2). If we schedule the kernels sequentially (as in a typical resource scheduler), we can run 10 threads of kernel I or kernel II, as the concurrency is determined by the largest single-resource consumption (e.g., 10% of resource B for kernel I). If the latency of running such threads is *T* for both kernels, this gives a throughput of 10/*T*. However, if we schedule both kernels concurrently, we could run 8 threads of both kernels I and II at the same time, leading to a throughput of roughly 16/*T*. Obviously, by scheduling kernels with complementary resource utilization patterns together, we avoid hitting the limit of a bottlenecking resource quickly. The problem can be very complex by considering more general cases with more resources and kernels involved.

**Fig 3:**
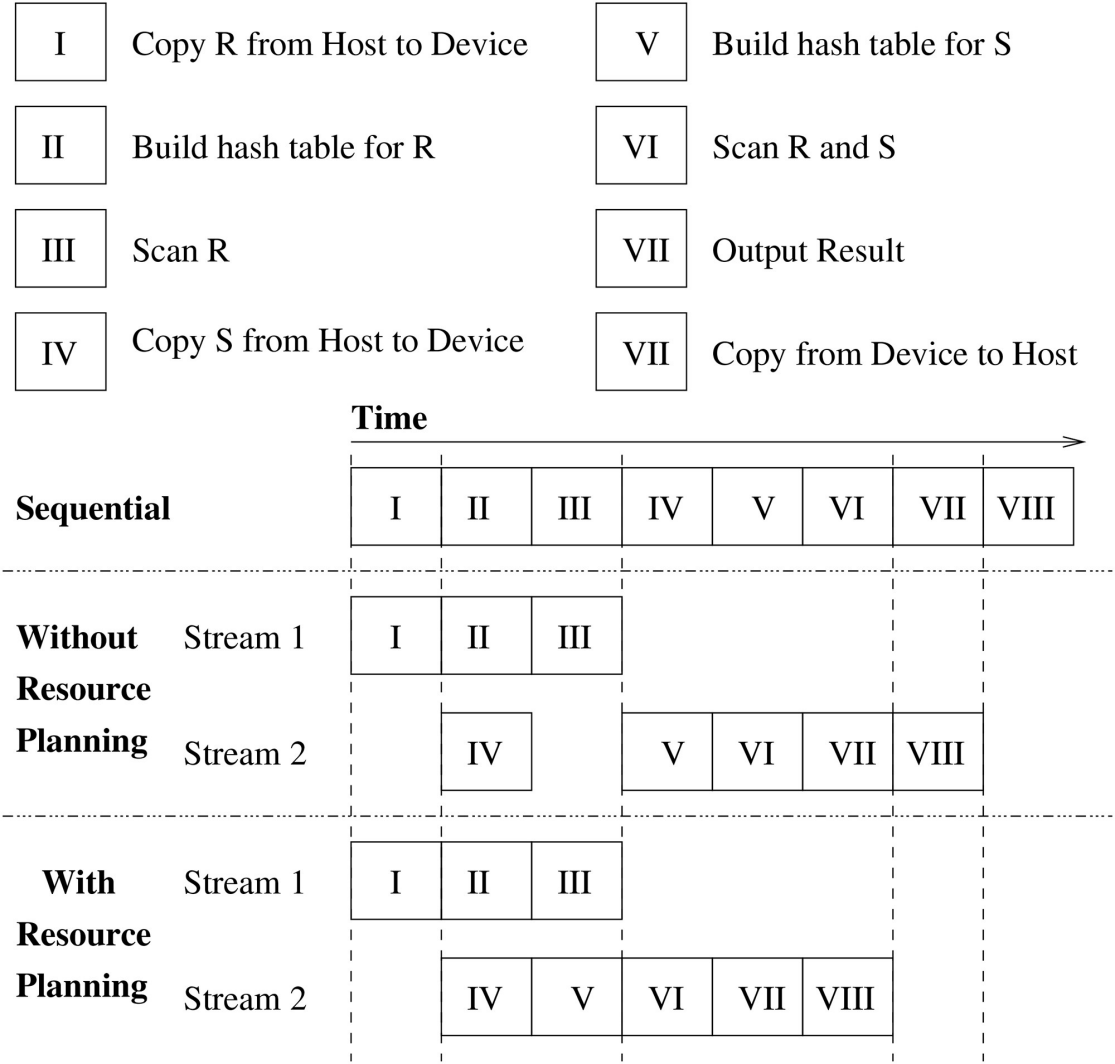
Three different schedules for launching kernels in a hash join between tables R and S.

The CUDA framework achieves hardware-level kernel concurrency via a mechanism named *CUDA Stream*. Our previous work [20] provides an in-depth anatomy of CUDA streams and identifies the scheduling disciplines of concurrent kernels. Given sufficient resources, many CUDA streams can run simultaneously with each stream containing one or more kernels. Fig 3 shows an example of hash join of table *R* and *S* (Query II mentioned before) with three different launching methods. Each rectangle presents a kernel, the kernel names are listed above. We can see if using CUDA stream with careful resource planning, building hash table of *R* (kernel II) and scan of table *R* (kernel III) can be completely parallel with copy table *S* from Host to Device (kernel IV) and building hash table of *S* (kernel V), therefore increasing throughput and reducing execution time. Note that, only kernels II, III, IV, and kernel V can be launched in parallel since other kernels have dependency with earlier ones. If using CUDA stream without resource planning, it can achieve partial concurrency but not optimal performance. Of course, if executing hash join without CUDA stream, the procedure would be sequential, which has the worst performance.

In CUDA, all threads in a block are scheduled to run on the same resource pool (i.e., the multiprocessor) thus a block can be conceptually viewed as a basic unit for studying our problem. On the other hand, CUDA allows a kernel to be launched with user-specified parameters and such parameters determine the actually resource use of each block of threads at runtime. Therefore, our problem essentially becomes: *how to set the runtime parameters of kernels in different CUDA streams to achieve the best throughput*?

To the best of our knowledge, optimization of multi-kernel parameters has not been studied before. As the first work on this topic, we aim at developing rigorous solutions under reasonable assumptions. Specifically, we develop an optimization model towards largest thread concurrency with the runtime parameters of all kernels as input. We identify the problem as a variation of the multi-dimensional Knapsack, which is a well-known NP-hard problem. A major contribution of our work is to simplify the original model such that efficient solutions are possible. In particular, via thorough analysis of the model structure and features of CUDA runtime system and CUDA streams, we reduce the number of dimensions of the constraints in the original model. As a result, we are able to develop an algorithm based on dynamic programming to solve the modified model. We prove the algorithm can find optimal solutions (in terms of thread concurrency) to the problem and bears pseudopolynomial complexity on both time and space. Such results are verified by extensive experiments running on our microbenchmark that consists of real-world and synthetic CUDA kernels. Furthermore, solutions identified by our method also significantly reduce the total running time of the workload, as compared to simple and random solutions. Similar to MultiQx-GPU, our work is more like a middleware to maximize the concurrency and minimized the modification of other tasks. It is natural to compare our work with MultiQx-GPU and the results are seen in ***Experimental Evaluation***.

### Paper organization

The remainder of this paper is organized as follows: we compare our study with related work in Section 1; in Section 2 we describe our optimization model and the analysis and simplification of the model; in Section 3, we present the dynamic programming algorithm; Section 4 describes experimental validation of our solution; we conclude the paper in Section 5.

## Related work

### Push-based database systems

In traditional DBMSs, the cost of I/O is expensive since its pull-based architecture needs to load data repeatedly. Sharing data among concurrent queries using a common I/O stream has become popular in database community. Harizopoulos *et al*. [21] enabled dynamic operator sharing with an on-demand simultaneous pipelining I/O system (OSP). Ramen *et al*. [22] implemented a system called Blink that runs every query based on a table scan. Frey *et al*. [23] designed an efficient join algorithm called cyclo-join to process queries under a distributed environment through a ring-structured network. Unterbrunner *et al*. [24] proposed a distributed relational table design called Crescando that uses shared scan to process data stream on multi-core machines. Another sharing data approach was studied in [25], which is based on a data-sharing model in both record and column disk storage. More recently, Arumugam *et al*. [9] developed a truly push-based system called DataPath, in which queries are pushed to processors and all the operations share data. This kind of push-based DBMS becomes the new trend in developing data management systems.

### GPGPU and databases

We focus on GPU as the platform because it provides much more computing power and lower energy consumption than modern CPUs. The advanced computing model such as CUDA [19] and OpenCL [26] accelerates its spread. It is very clear that it becomes a popular computational platform in many application domains [27, 28]. The data management community has also done a lot of work on improving database performance using GPUs. GPU-based algorithms for computing major relational operators were developed by Govindaraju *et al*. [29], who reported dramatic performance improvement over a compiler-optimized SIMD implementation with up to 40 times speedup. Bakkum *et al*. [30] implemented a subset of command processors based on the open-source database named SQLite to achieve GPU acceleration. Pinnecke *et al*. [4] presented a variable-length window in stream processing of DBMS on GPUs. Sitaridi *et al*. [31] proposed a bank optimization solution for improving data access performance on GPU memory. It focused on resolving the conflict issues when using shared memory on GPUs in order to fully utilize the bandwidth of shared memory therefore enhancing performance. And there are works about improving join algorithms on GPU. He *et al*. [32] implemented novel relation join GPU algorithms that obtained 2-7X better performance as compared to CPU-based algorithms. Kaldewey *et al*. [33] implemented some join processing algorithms on GPUs, and they got a 50% performance boost over CPU implementations of the same algorithms. Ran *et al*. [34] revisited He’s algorithm after seven years under modern GPU architecture and achieved up to 20X speedup over the CPU-based join algorithms and [35] developed an fast Equi-Join algorithms on GPU later. As for implementing the integrated GPU-based DBMS, Yuan *et al*. [36] developed a query engine that adopts a block-oriented execution model which executes a given query plan tree in post-order sequence. Zhang *et al*. [16] proposed a kernel-adapter GPU-based DBMS called OmniDB that can put cost model, execution engine, and scheduler all together into a hardware oblivious database kernel (qkernel) to maximize the common functionality in qKernel, in this way the development and maintenance costs are minimized. Wu *et al*. [18] developed a compiler and runtime infrastructure called Red Fox to execute relational queries on GPUs. Paul *et al*. [5] implemented a novel pipelined query execution engine called GPL for query co-processing on the GPU. In industry, MapD and Kinetica are two companies that leads in GPU-based DMBS system domain. MapD and Kinetica are both in-memory GPU-accelerated distributed databases that combines query processing with analytic and visualization functionalities [7, 8].

### GPGPU performance modeling

Besides database community, other research domains studied performance modeling on GPUs. Xu *et al*. [37] proposed a GPU-accelerated simulation model for high-fidelity network systems. Baghsorkhi *et al*. [38] presented an analytical model to predict the performance of GPU applications with the help of an abstract interpretation method called work flow graph. Hong *et al*. [39] proposed an analytical model that estimates the execution time of programs running on GPUs and an improved version [40] later. The model estimates the number of parallel memory requests via analysis of program behavior and instructions. The same research group [41] also developed an empirical power model for GPUs. Kerr *et al*. [42] introduced a model based on Hong’s analysis to predict relative performance of the same applications running on GPUs and CPUs. However, all above modeling efforts focused on single-kernel tasks on GPUs and single-kernel modeling efforts are not readily applicable to simultaneous multi-kernel scenarios. Moreover, the modeling methods mentioned above either require extra effort to achieve accurate prediction or focus on a specific domain that is not applicable to our problem. This is also the motivation to conduct our research in this paper.

### Multi-tasking in GPUs

Guevara *et al*. [43] proposed a queueing system that schedules and merges CUDA kernels within one kernel to achieve task parallelism. Li *et al*. [44] analyzed the factors affecting the parallel execution performance on GPUs and conducted a theoretical performance estimation. With the help of Dynamic Parallelism in CUDA (a feature enables the launch of parallel work at runtime on a GPU), Krieder *et al*. [45] proposed an execution model and run-time system called GeMTC to decompose kernels into warp-level and integrated with Swift language. This proposal requires rewriting user kernels (i.e., decomposing into warp-level units) while kernels are treated as atomic units in our scheme based on CUDA streams. Wang *et al*. [46] adopted Kernel Preemption (a technique that can swap the context of a kernel on one SM with the context of a new kernel) and developed a dynamic sharing mechanism named Simultaneous Multikernel (SMK) by improving resource utilization to boost performance. This technique is meant to be implemented in the GPGPU runtime system and only evaluated in a simulator while our strategy runs at the middleware level and is fully tested in a real system. Those work use alternative approaches to achieve concurrency or partial concurrency, while our work addresses on concurrent kernel execution and resource allocation with CUDA stream to achieve real-time concurrency.

Some of the GPU databases research mentioned above also involve multi-kernel execution, such as Red Fox [18] and GPL [5]. Both systems analyze query and generate a new query plan so that in the query, operators that are unrelated to others can be executed in a concurrent manner. The difference between Red Fox and GPL is that GPL has more optimized features such as tile-based pipelined execution model and data channel. However, both methods focused on improving the performance of single query by parallelizing individual database operators, none of them can really handle multi-query environment. Unlike them, MultiQx-GPU [17] is the only one that supports multi-query processing in GPU, this is the reason why we chose it as the baseline to compare and we will introduce it in the following paragraph.

Besides studying the multitasking on system level, there are also some researches of multi-tasking in GPU on application level. Park *et al*. [47] proposed GPU Maestro to maximize multi-tasking performance in GPU by dynamically manages resource partitioning. Liang *et al*. [48] developed a new cache management mechanism for multi-tasking on GPUs. Sorensen *et al*. [49] proposed cooperative kernels of blocking algorithms that can support multitasking. Nouri *et al*. [50] presented a framework named G-PICS for parallel searches. Zhao *et al*. [51] used Classification-Driven search for low-overhead dynamic SM partitioning to enable multitasking in GPU.

### Overview of MultiQx-GPU

MultiQx-GPU [17] supports concurrent query processing by enabling GPU resource sharing among database queries. The design of MultiQx-GPU follows two main principles: versatility and high efficiency. Versatility means the system is applicable to different GPU databases and GPU computing frameworks (e.g., CUDA, OpenCL, and DirectCompute). High efficiency is credited to the multitasking of MultiQx-GPU. It support multitasking (main part of it is overlapped data transfer between GPU and CPU) by implementing system-level functions similar to the concept of CPU-based systems like virtual memory (VM) and fine-grained context switches. In this way, high overhead of copying data between devices can be reduced.

**Fig 4:**
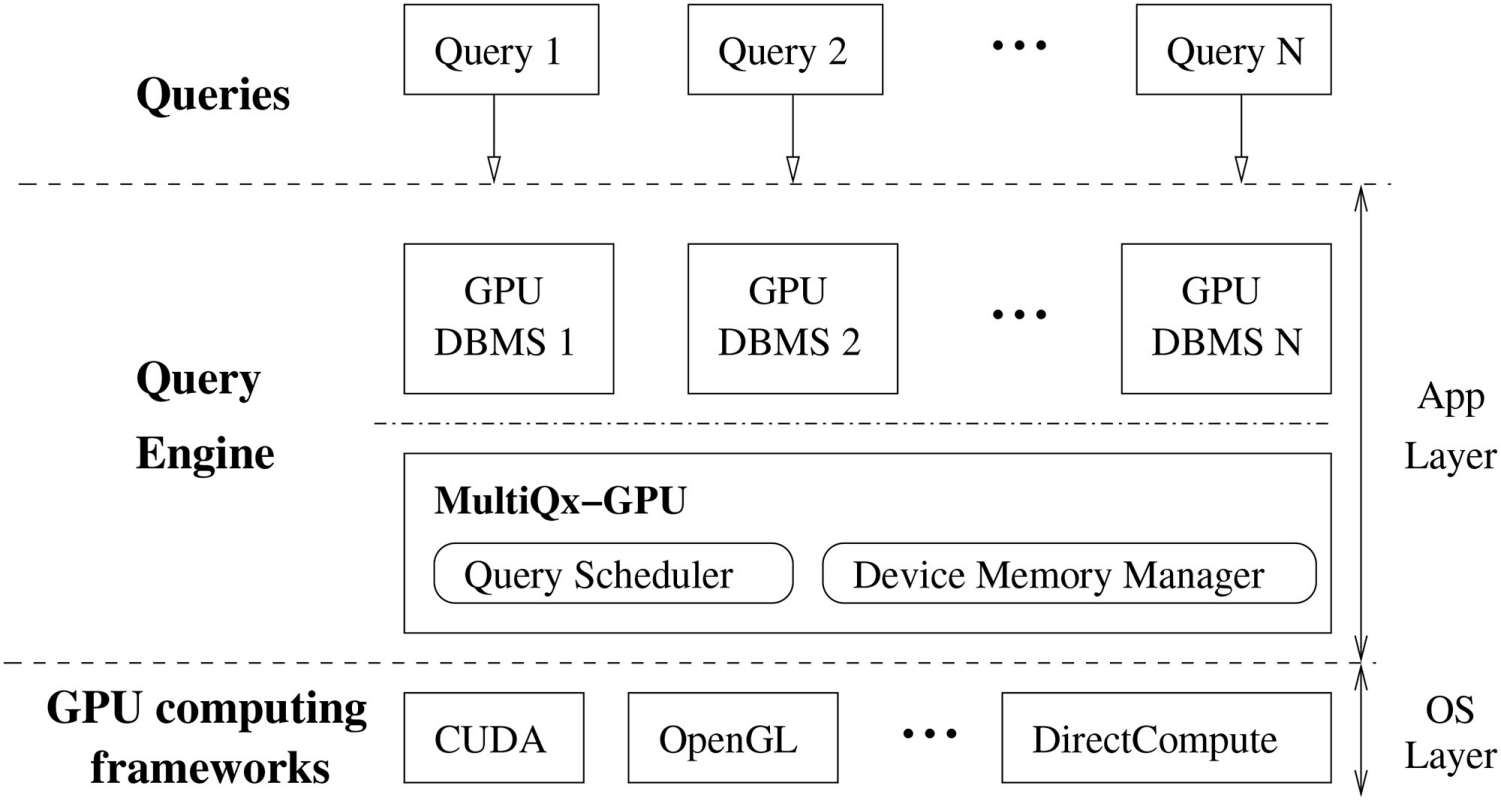
Overview of MultiQx-GPU [17].

Fig 4 shows the architecture of MultiQx-GPU as well as its position in an multitasking GPU environment. MultiQx-GPU is built in Query Engine and serves as a middle layer between existing GPU DBMSs and GPU computing frameworks. It takes over GPU resource usage by intercepting GPU API calls related to resource management. It does not change the query engine algorithm of existing GPU DBMSs and the existing programming interfaces of GPU drivers. Thus MultiQx-GPU can be easily deployed between different GPU DBMSs and GPU computing frameworks. Query Scheduler and Device Memory Manager, the two main components of MultiQx-GPU, completely resides on Application Layer, thus they don’t rely on any OS-layer privileges of GPU computing frameworks. Query scheduler controls concurrency level by maintaining the optimal workload on the GPUs, which means it only allows queries that can execute concurrently to run on GPU, in this way it can maximize system throughput. Device Memory Manager dissolves the resource conflicts of concurrent queries by overlapping memory allocation and data transferring with VM-like automatic data swapping service, it further enhances the performance.

## Multi-kernel optimization

In this section, we present our modeling and control of concurrent tasks in a multi-kernel GPGPU environment. Firstly, we briefly introduce modern GPU architecture. Next, we illustrate the overview of our approach. Then, we present the development of our Kernel-level Optimization Model as well as its analysis and simplification. Finally, we bring out our Batch-level Optimization Model.

### Typical GPU architecture

**Fig 5:**
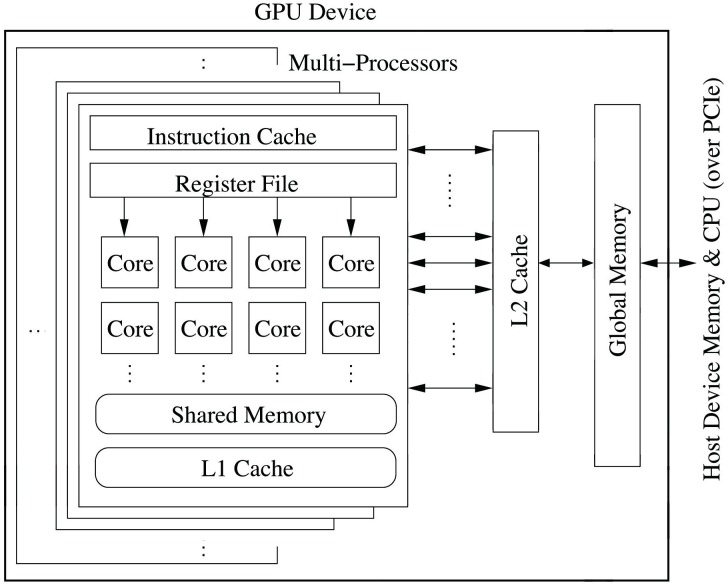
Architecture of a typical NVidia GPU [20].

A modern GPU is a special hardware that encapsulates many processing units together to provide high parallel computing capability. As shown in Fig 5, main components of a GPU includes: (1) A number of *Multi-Processors* (MP) that each groups tens of processor cores together. The cores execute threads in a Single-Instruction-Multiple-Data (SIMD) fashion; (2) *Multi-level memory*. Of largest amount (e.g., 12GB for the Titan X Pascal) is the *global memory*, which can be accessed in parallel by cores in different MPs. The bandwidth of global memory can be as high as 480GB/s [52]. GPU also offers high-speed on-chip cache called *shared memory* (SM) similar to L1 cache, and each MP has its own SM with a size up to 96KB [53]. SM is user programmable in GPU code and is not visible to the CPU code. Within each MP, there is also other memory: the read-only data cache 24 KB [53] and the nonprogrammable L2 cache with a certain size (3 MB) and a bandwidth smaller than that of SM [54].

In the CUDA programming framework, a function to be executed in a parallel way is called a CUDA *kernel*. A kernel can be spawned with a large number of computational *threads*. Threads for a kernel is called a *grid* and the grid is divided into *blocks* that each contains the same number of threads to be executed on a single MP. On the other hand, multiple blocks can be run on the same MP, and one MP can process up to 32 blocks. It is device driver’s responsibility to schedule the blocks to use the different MPs. Threads are scheduled as groups of 32 threads called *warps*. The entire global memory can be accessed by any thread in any MP, shared memory and registers of each MP can only be accessed by the thread of the same MP. CUDA provides a mechanism called *CUDA stream* with the ability to schedule multiple CUDA kernels simultaneously. One CUDA stream can encapsulate multiple kernels, and they have to be scheduled strictly following a particular order. However, kernels from multiple streams can be scheduled to run concurrently. However, NVidia does not reveal much detail about the internal mechanism for kernel scheduling in CUDA streams. Our previous work [20] studied kernel scheduling policies of CUDA streams, the findings of that work form the foundation of this paper.

### Overview of our approach

MultiQx-GPU achieves multi-kernel concurrency by controlling the workload from CPU side and overlapping the I/O between CPU and GPU. In other words, kernel execution on GPUs is still sequential for MultiQx-GPU. In contrast to that, our approach focuses on resource sharing among different kernels in GPUs.

A GPU contains different types of resources including physical hardware units and software constraints. In our previous work [20], we have identified three types of resources / constraints that affect the performance in a single-kernel setup: *registers*, *shared memory*, and *maximum warps allowed in an MP*. In a multi-kernel environment, there is one additional constraint we have to consider: *total blocks of all the kernels allowed to run simultaneously in an MP*.

As long as all the resources are sufficient, multiple kernels can be executed at the same time. For any kernel, its resource consumption can be controlled at runtime by changing the launching parameters in the host (CPU) code. CUDA allows three parameters in launching a kernel: *total number of blocks*, *block size* (i.e., number of threads in a block), and *shared memory consumption* as an optional parameter. Note that the product of the first two is actually the total number of threads. The third parameter is generally not specified, as programmers often hardcode the total shared memory use to match the size of a chunk of input data. Therefore, in this paper, we only consider *total number of blocks* and *block size* as the controls we apply to affect resource consumption. Note that, in CUDA, each thread gets its own set of registers while the shared memory is shared by all threads in the block. Therefore, by changing the block size, we can control the register use per block and shared memory use among all blocks of a kernel. Needless to say, the block size itself directly determines the number of warps per block.

Before we start developing our optimization model, it is worth mentioning that the problem of optimizing single-kernel performance was solved in our previous work [20]. In particular, we build a model to quantify the total number of threads that can be executed simultaneously (i.e., *occupancy* in CUDA terminology) as an indication of kernel performance. Based on this model, we can accurately predict kernel performance under any block size and then pick the one with highest performance to run. Although some ideas can be borrowed, the same problem under a multi-kernel environment is much more complicated. First, the modeling method based on a series of discrete functions for the single-kernel situation will only yield models that are too complicated to handle; Second, kernel scheduling rules among different CUDA streams are not revealed by NVidia—such information is vital for the development of our optimization model; Last, with multiple kernels, the solution space of the optimization problem increases exponentially. This places stringent requirements on the efficiency of the algorithm(s) for solving the optimization.

**Fig 6:**
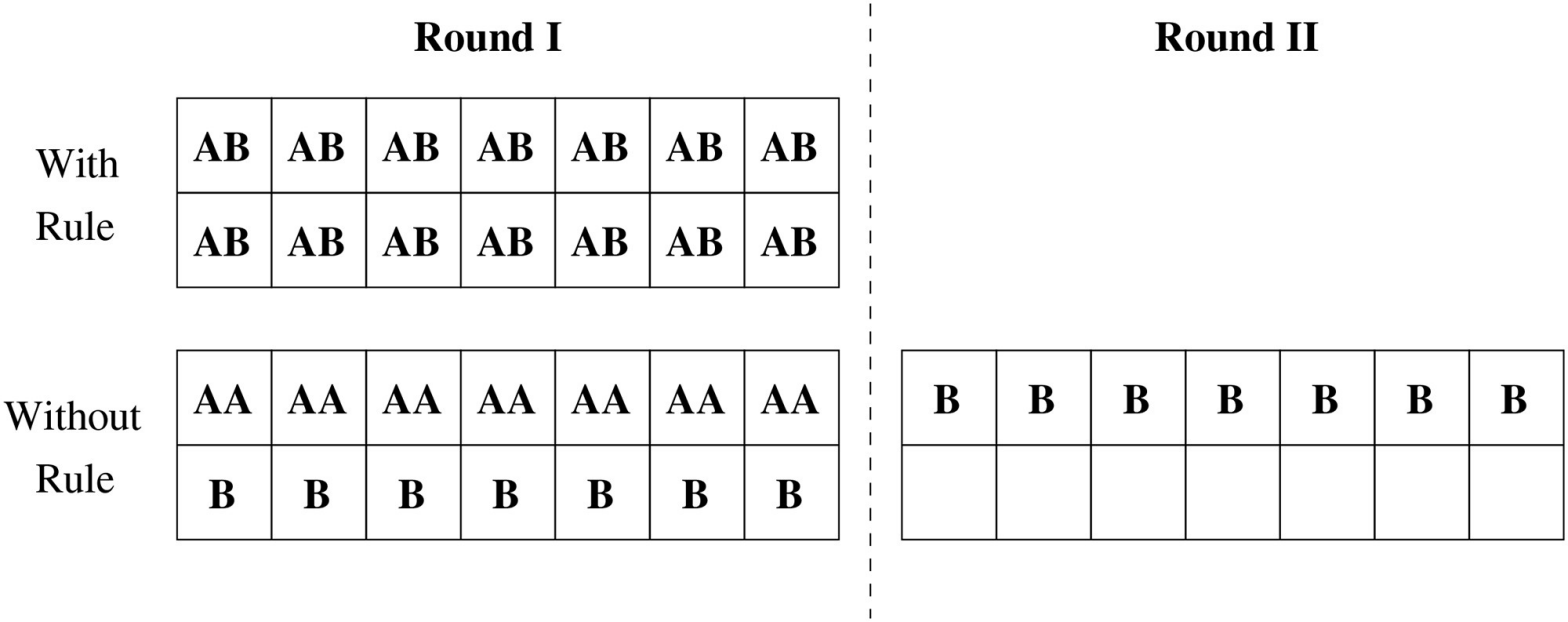
Two different ways to schedule two CUDA kernels, each of which is in a CUDA stream.

That said, our previous work [20] also built a solid foundation for multi-kernel modeling by identifying basic rules of CUDA stream scheduling. Here we briefly present one scheduling rule that is most relevant to our modeling. The rule says: *CUDA scheduler always takes as many MPs as possible in scheduling the different blocks of a kernel*. For example (Fig 6), if we have two kernels A and B, both of them have 14 blocks, and there are 14 MPs. Each MP can run two blocks of A, or one block of B, or one block of A and one block of B at the same time. Based on the rule above, CUDA scheduler will schedule one block of A to each MP, then schedule one block of B to each MP, now there are one block of A and one block of B running on each MP. Without the rule, the scheduler will put as many as blocks of A into MP, which makes each of seven MPs has two blocks of A, and each of the rest seven MPs has one block of B, while the rest of seven blocks of B needs to wait for the next round to be scheduled.

According to the above rule of CUDA stream scheduling, our model can target one MP, the final result of each kernel is the number of MPs times optimized blocks. In particular, we divide the total threads of each kernel by the number of MPs, using the result as the total thread in our model. In this way, we make sure each MP has same amount and portion of kernels. We also assume that *there is at least one solution for all the kernels to fit in the MPs*. Otherwise, the left kernels need to wait for another round to run. If there is a situation that combined MPs can hold the total threads of all the kernels while a single MP cannot (i.e. the number of kernels exceed the maximum number of blocks in an MP), we group two MPs as a unit, which means we divide the total threads of each kernel by half number of MPs.

### Kernel-level optimization model development

The desirable optimization goal of the multi-kernel resource allocation problem is total running time of all kernels. However, it is difficult (if possible at all) to derive a model that maps the launching parameters of multiple kernels to running time. This is mainly due to the lack of low-level details of CUDA runtime environment. To the best of our knowledge, no one has done research on performance modeling in a real multi-kernel GPU environment. In this paper, we set the optimization goal to be *maximizing concurrency*, which is defined as the total number of threads that can be scheduled to run at the same time. Such a goal is meaningful for two reasons: (1) it is a direct measurement of throughput; and (2) as shown in our previous research [20], concurrency has a strong (negative) relationship with kernel running time.

To achieve maximum concurrency on a GPU, we need to get the most threads (of different kernels) running in an MP (Eq (1)). The problem can be formulated as the following integer programming statement:

$$\begin{array}{cc} \text{Maximize} & \;\;\sum_{i}\sum_{j}32jx_{ij}b_{i}\; \end{array}$$ (1)

$$\begin{array}{cc} \text{subject}\;\text{to} & \;\;\sum_{i}\sum_{j}32jx_{ij}b_{i}r_{i}\leq R\; \end{array}$$ (2)

$$\begin{array}{cc} & \;\;\sum_{i}\sum_{j}jx_{ij}b_{i}\leq W\; \end{array}$$ (3)

$$\begin{array}{cc} & \;\;\sum_{i}b_{i}s_{i}\leq S\; \end{array}$$ (4)

$$\begin{array}{cc} & \;\;\sum_{i}b_{i}\leq B,b_{i}\in Z_{+}\; \end{array}$$ (5)

$$\begin{array}{cc} & \;\;\sum_{j}x_{ij}=1,\forall i\; \end{array}$$ (6)

$$\begin{array}{cc} & \;\;\sum_{j}32jx_{ij}b_{i}\geq c_{i},\forall i\; \end{array}$$ (7)

$$\begin{array}{cc} & \;\;x_{ij}\in \left\{0,1\right\},\forall i,j\; \end{array}$$ (8)

In the above statement, *i* is the index of a kernel, *j* is the index of all the possible choices of block size for a single kernel. Since CUDA schedules 32 threads (a warp) as a unit, we use warp instead of thread in this model, 32*j* stands for number of threads in a block for a single kernel. To be specific, CUDA allows a block to have up to 32 warps in it therefore we have *x*_*i*,*j*_ = *j* (*j* ∈ [1, 32]), *x* is a binary number to represent which block size is chosen in a solution (Eqs (6) and (8)). The quantities *b*_*i*_ and *s*_*i*_ stand for the number of blocks and shared memory use for kernel *i*, respectively. *r*_*i*_ is the per-thread numbe of register for the same kernel. The constants *R*, *W*, *S*, and *B* stand for the total number of registers, warps, shared memory and blocks of an MP in the GPU. The reason for having Eq (7) is as follows: for most CUDA programs, the total number of threads *c*_*i*_ is fixed by the programmer to cater to the data size, changing total blocks and block size are actually the same: when block size increases by a factor of *f*, total number of blocks will decrease by the same factor *f*. However, the data size of a kernel can hardly be a multiplier of 32, thus we use ≥ instead of = in Eq (7). For each kernel *i*, *r*_*i*_, *c*_*i*_, and *s*_*i*_ are constants thus the inputs to the optimization problem. On the other hand, the solution to the optimization contains quantities *x*_*ij*_ and *b*_*i*_.

**Remark**:

1. Note that the aforementioned formulation has an interesting feature: according to Eq (7), any **feasible** solution to the formulation actually provides us a schedule with the maximal concurrency. However, due to a large number of 0 − 1 variables (*x*_*ij*_ for all *i* and *j*) and the other 6 non-trivial constraints, it is an NP-hard problem to locate a feasible solution. To address such challenges, we discuss model simplification and transformation in next subsection. Such transformation results in the development of a pseudo-polynomial algorithm in solving the problem (***Algorithm of Kernel-optimization model***);
2. Via Eq (7), we made an assumption that solutions to the formulation do exist. In other words, we can find a set of launching parameters for every kernel such that they can all be processed by the GPU at the same time. In ***Batch-level Optimization Kernel for More a General Situation***, we briefly discuss a more general version of the problem with this assumption removed.

### Kernel-level optimization model analysis and simplification

By studying the structure of the current model, we realize it is a flavor of the well-known *multidimensional knapsack problem* (MKP). An MKP is NP-hard even when the number of constraints is only one [55]. It is easy to see our model is equivalent to a **four** MKP therefore it is also an NP-hard problem. Moreover, our model involves a binary variable *x*_*ij*_ as part of the solution and as many as seven constraints. Therefore, the original formulation is difficult to analyze or to compute. To remedy that, we aim to transform the model into a form that is easier to handle via considering the actual environment where our problem is defined. Specifically, we derive a reformulation with a much smaller number of variables and constraints.

Our first goal is to eliminate the binary integer *x*_*ij*_. As mentioned before, CUDA schedules threads in groups of 32 (i.e., a warp). For example, if we launch a kernel with 240 threads, the CUDA runtime framework will actually launch 8 warps for this kernel (with the last warp containing empty threads in this case). Therefore, we use warp number *w*_*i*_ ($w_{i}=\frac{t_{i}}{32}$) to replace *jx*_*ij*_, and the value of *w*_*i*_ ranges from 1 to 1024/32 = 32 (since the maximum block size is 1024). As a result, the total number of threads of a kernel has a ceiling of the total threads in the assigned warps, Eqs (3) and (7) become:

$$\begin{array}{cc} & \sum_{i}w_{i}b_{i}\leq W \end{array}$$ (9)

$$\begin{array}{cc} & \;\;32w_{i}b_{i}\geq c_{i},\forall i\; \end{array}$$ (10)

We then aim at removing some of the constraints. As we mentioned, any feasible solution to the original model is actually a solution that gives us the maximal concurrency. Hence, it suffices to develop a new model that aims at finding one feasible solution to the original model with fewer constraints. Note that based on Eq (10), we can easily calculate the results of ∑_*i*_∑_*j*_ 32*jx*_*ij*_ *b*_*i*_ *r*_*i*_ given any problem inputs. Thus, the constraint about registers in Eq (2) only serves the purpose of determining if there is a feasible solution, and we can remove it from the problem statement. Now we have the newly derived constraints shown in Eqs (9) and (10) plus the remaining constraints Eqs (4) and (5).

With the above constraints, we further reduce the level of difficulty in solving the problem via a technique that modifies the objective function. This can be done by transforming a constraint into the object function. In particular, we can choose any of the remaining constraints as our new object function. In our problem, we pick Eq (4) since it is the only one that has a unique coefficient *s*_*i*_. Consequently, the new problem formulation becomes:

$$\begin{array}{cc} \text{Minimize} & \sum_{i}b_{i}s_{i} \end{array}$$ (11)

$$\begin{array}{cc} \text{subject}\;\text{to} & \sum_{i}b_{i}\leq B \end{array}$$ (12)

$$\begin{array}{cc} & \sum_{i}w_{i}b_{i}\leq W \end{array}$$ (13)

$$\begin{array}{cc} & 32w_{i}b_{i}\geq c_{i},\forall i \end{array}$$ (14)

$$\begin{array}{cc} & b_{i}\in Z_{+}\;\forall i. \end{array}$$ (15)

**Remark**: The new problem has the following two features:

1. *Equivalence*: if the optimal value of the new formulation is less than or equal to total shared memory *S*, the corresponding optimal solution is feasible to the original formulation. According to the first remark made after Eqs (1)–(8), in this case we actually find a schedule with the maximal concurrency;
2. *Simplicity*: although this reduced formulation deals with general integer variable *b*_*i*_, we have way fewer discrete variables, along with only three non-trivial constraints, which indicates its computational burden might not be heavy in practice, if a well-designed algorithm can be developed. Note that the quantities *b*_*i*_ and *w*_*i*_ are the solutions and all other quantities are inputs to the model.

After a series of transformations without adding new assumptions, the problem becomes one to *minimize the total shared memory use of all kernels*. This is intuitive, as minimizing shared memory use of one kernel will also minimize its number of blocks so that there are more space left for remaining kernels in dimension of *B* (see Eq (4) in original model).

### Batch-level optimization kernel for more a general situation

As we mentioned, our model assumes all kernels can fit in an MP. However, there could be more general scenarios in which an MP cannot accommodate all kernels due to resource constraints. For such problems, our solution is to run all the kernels in different batches, each batch will fit in an MP. In each batch, we solve the above model to get a batch-level solution. Then the key problem becomes *how to determine the membership of each batch*. Specifically, the problem can be formulated as follows:

$$\begin{array}{cc} \text{Minimize} & G=\sum_{k}y_{k} \end{array}$$ (16)

$$\begin{array}{cc} \text{subject}\;\text{to} & G\geq 1 \end{array}$$ (17)

$$\begin{array}{cc} & \sum_{i}w_{i}x_{ik}\leq W_{y_{k}} \end{array}$$ (18)

$$\begin{array}{cc} & \sum_{i}r_{i}x_{ik}\leq R_{y_{k}} \end{array}$$ (19)

$$\begin{array}{cc} & \sum_{i}s_{i}x_{ik}\leq S_{y_{k}} \end{array}$$ (20)

$$\begin{array}{cc} & x_{ik}\in \left\{0,1\right\},\forall i,k \end{array}$$ (21)

$$\begin{array}{cc} & y_{k}\in \left\{0,1\right\},\forall k \end{array}$$ (22)

In this problem, we still target the maximum concurrency, i.e., we want to pack as many kernels as possible in a batch, thus the number of batches *G* is minimized, as shown in Eq (16). Each *G* has the same maximum capacity, *i.e.*, total warp numbers *W*, register numbers *R*, and shared memory *S*. Here, *k* is the index of a batch, *y*_*k*_ is a binary variable where *y*_*k*_ = 1 if bin *k* is used, and *i* is the index of a kernel, *x*_*ik*_ is a binary variable setting to 1 if kernel *i* is put in batch *k*. Same as the model described earlier, the quantities *r*_*i*_, *w*_*i*_ and *s*_*i*_ stand for the register number (per thread), warp number, and shared memory use for kernel *i*, respectively. For each kernel *i*, *w*_*i*_, *r*_*i*_, and *s*_*i*_ are constants thus the inputs to the optimization problem, the solution to the optimization problem contains quantities *x*_*ik*_.

The above pre-processing model is a three-dimensional Bin Packing Problem (3D-BPP), which is strongly NP-hard [56]. Silvano *et al*. [57] proved that the lower bound of Bin Packing Problem is $\frac{1}{8}$, which is the asymptotic worst-case performance. We will introduce the algorithm in ***Algorithm of Batch-optimization model***.

## Solving the optimization problem

In this section, we present algorithms to focus on solving the kernel-optimization model shown in Eqs (11) to (15) firstly, then presenting the algorithm of the batch-optimization model shown in Eqs (16) to (22).

### Algorithm of Kernel-optimization model

In this section, we present algorithms to solve the simplified model of kernel-level shown in Eqs (11) to (15). Note that the new formulation is not a simple knapsack problem anymore. Indeed, because both *w*_*i*_ and *b*_*i*_ are variables, the formulation in Eqs (11)–(15) is a *quadratic general knapsack problem* (QGKP), which is also an NP-hard problem [58]. Hence, a brute-force algorithm would have to search through all *O*(*BW*) possible combinations with respect to a total of *n* kernels, giving a total time complexity of *O*((*BW*)^*n*^), and this is clearly infeasible for practical instances.

However, the transformation of the original problem into QGKP enabled us to develop a (practically) efficient algorithm based on the *dynamic programming* approach. Dynamic Programming is a well-known divide-and-conquer technique to solve optimization problems. The idea is to transform a complex problem into relatively simple sub-problems. The algorithm examines previously solved sub-problems and combine the solution to give a best solution for a slightly larger sub-problem.

Applying dynamic programming to knapsack problem is to essentially trade time with space. We can use a table to record decisions made for sub-problems and recursively look up the table when involving previous decision. Following our discussions in ***Kernel-level Optimization Model Analysis and Simplification***, we should use a three-dimensional table since there are three variables to be considered: the *n* kernels, total blocks ranging from 0 to *B*, and total warps ranging from 0 to *W*. The main task of the algorithm is to compute the value of a cell (*i*, *b*, *w*) in this table, where *i* is the kernel number, *b* is the block number of kernel *i*, and *w* is the warp number of kernel *i*, respectively. Here cell value (*i*, *b*, *w*) stands for the minimum total shared memory used of any subset of kernels 0 to *i* under targeted block number *b* and targeted warp number *w*. The key feature of the algorithm is that we only need to consider local choices in the table. In particular, the following result helps us drastically reduce the complexity of the table.

**Theorem 1**. *For a particular kernel i, if b*_*i*_ *is fixed, an optimal choice of w*_*i*_ *can be obtained as* $w_{i}=\lceil \frac{c_{i}}{32b_{i}}\rceil$.

*Proof*. Note that to satisfy Eq (14), *w*_*i*_ must be greater than or equal to $\left\lceil \frac{c_{i}}{32b_{i}}\right\rceil$. Also, the smaller *w*_*i*_, the smaller left-hand-side of Eq (13). So, it would be optimal to set $w_{i}=\lceil \frac{c_{i}}{32b_{i}}\rceil$.

Hence, in the remainder of this paper, we simply set $w_{i}=\lceil \frac{c_{i}}{32b_{i}}\rceil$ when *b*_*i*_ is available. Moreover, our dynamic programming algorithm can be simplified into a form similar to that for the general knapsack problem. Specifically, let *V* [*i*, *b*, *w*] be the objective value considering up to *i*-th kernel with total *b* blocks and *W* warps. The Bellman equation is

$$\begin{array}{c} V\left[i,b,w\right]=min_{b_{i}=1,\ldots ,B}\left\{V[i-1,b-b_{i},w-w_{i}b_{i}]+s_{i}b_{i}\right\} \end{array}$$

where *s*_*i*_ is shared memory usage per block of kernel *i*. Note that whenever *b* or *w* causes the solution infeasible, we will set the corresponding *V* to ∞.

**Algorithm 1**: Kernel-level Optimization Algorithm

1: **for** *b* ← 0 to *B* **do**

2:  **for** *w* ← 0 to *W* **do**

3:   *V*[0, *b*, *w*] ← 0

4:   *P*[0, *b*, *w*] ← *ϕ*

5:  **end for**

6: **end for**

7: **for** *i* ← 1 to *n* **do**

8:  *V*[*i*, 0, 0] ← ∞

9:  *P*[*i*, 0, 0] ← *ϕ*

10: **end for**

11: **for** *i* ← 1 to *n* **do**

12:  **for** *b* ← 1 to *B* **do**

13:   *Q*_*b*_ ← *V*[*i* − 1, *B* − *b*, *W* − *wb*] + *s*_*i*_ *b*

14:  **end for**

15:  *V*[*i*, *b*, *w*] ← *min*_*b* = 1,…,*B*_{*Q*_*b*_},

  /* denote optimal *b* as b* */

16:  *P*[*i*, *b*, *w*] ← *P* [*i* − 1, *B* − b*, *W* − *w*_*i*_ b*] ∪ (*i*, b*)

17: **end for**

Details of the algorithm to solve our problem can be seen as pseudocode in Algorithm 1. After we compute all the entries of *V*, *V*[*n*, *B*, *W*] will contain the minimum shared memory use achieved by the solution. Meanwhile, another array *P* holds the solutions to the sub-problems and *P*[*n*, *B*, *W*] is our solution. With the principle of optimality carried in the general knapsack problem, the correctness of the algorithm is shown as follows.

**Theorem 2**. *Algorithm 1 terminates with an optimal solution, i.e., the value of V*[*n*, *B*, *W*] *is optimal*.

*Proof*. We prove the theorem via induction.

1. When there is one kernel (*n* = 1), we have
  $$\begin{array}{ccc} V[1,1,w] & = & min\{V[1,1-1,w],\\ & & V\left[0,B-1,W-w\right]+s_{1}\}\\ & = & min\left\{\infty ,0+s_{1}\right\}=s_{1} \end{array}$$
  For *V*[1, 1, *w*], we get the optimal value *s*_1_.
  $$\begin{array}{ccc} V[1,2,w] & = & min\{V[1,2-1,w],\\ & & V\left[0,B-2,W-w\right]+2s_{1}\}\\ & = & min\{s_{1},0+2s_{1}\} \end{array}$$
  For *V*[1, 2, *w*], we can get the optimal value by comparing *V*[1, 1, *w*] and 2*s*_1_.
  $$\begin{array}{ccc} V[1,B,w] & = & min\{V[1,B-1,w],\\ & & V\left[0,B-B,W-w\right]+s_{1}B\} \end{array}$$
  If we know the optimal value of *V*[1, *B* − 1, *w*], we can get the optimal value of *V* [1, *B*, *w*] by comparing *V* [1, *B* − 1, *w*] and 0 + *B* × *s*_1_. Deriving it one by one, we can get the optimal value of *V*[1, 1, *w*], then value of *V*[1, 2, *w*] based on *V* [1, 1, *w*], ⋯, and value of *V*[1, *B*, *w*] based on *V*[1, *B* − 1, *w*]. Thus for each *b* from 1 to *B*, we get the optimal value.
2. When there are two kernels (*n* = 2), we have
  $$\begin{array}{ccc} V[2,1,w] & = & min\{V[2,1-1,w],\\ & & V\left[1,B-1,W-w\right]+s_{2}\times 1\}\\ & = & min\{\infty ,V\left[1,B-1,W-w\right]+s_{2}\}\\ & = & V\left[1,B-1,W-w\right]+s_{2} \end{array}$$
  From Step (1) we know *V*[1, *B* − 1, *W* − *w*] has an optimal value, so *V*[2, 1, *w*] has the optimal value.
  $$\begin{array}{ccc} V[2,2,w] & = & min\{V[2,2-1,w],\\ & & V\left[1,B-2,W-w\right]+s_{2}\times 2\}\\ & = & min\{V\left[2,1,w\right],V\left[1,B-2,W-w\right]+2s_{2}\} \end{array}$$
  Also from step (1) we know *V*[1, *B* − 2, *W* − *w*] has an optimal value, and *V*[2, 1, *w*] has optimal value based on above proof. By comparing *V*[2, 1, *w*] and *V*[1, *B* − 2, *W* − *w*] + *s*_2_ × 2 we can get the optimal value of *V*[2, 2, *w*].
  $$\begin{array}{ccc} V[2,B,w] & = & min\{V[2,B-1,w],\\ & & V\left[1,B-B,W-w\right]+s_{2}\times B\} \end{array}$$
  Same as in step (1), we derive it one by one, we can get the optimal value of *V*[2, 1, *w*], then value of *V*[2, 2, *w*] based on *V*[2, 1, *w*] and *V*[1, *B* − 1, *W* − *w*], ⋯, and value of *V*[1, *B*, *w*] based on *V*[2, *B* − 1, *w*] and *V*[1, *B* − *B*, *W* − *w*]. Thus for each *b* from 1 to *B*, we can get the optimal value.
3. The same approach shown in step (2) can be applied to cases *n* = 3 and beyond, and this concludes the proof.

#### Time and space complexity

The complexity is clearly determined by the size of the dynamic programming table, which is *O*(*nBW*). In practice, both *n* and *B* are small integers (i.e., *n* ≤ 32 and *B* ≤ 16 in the latest version of CUDA) thus this algorithm will have negligible cost. Similarly, the pre-processing stage takes *O*(*nBW*).

### Algorithm of Batch-optimization model

The 3D-BPP is a hardly NP-hard problem, to our knowledge no one has present an exact algorithm for it. We have developed a solution by applying classic Gilmore and Gomory algorithm [59] [60] after transforming this problem into a cutting stock problem. While the Gilmore and Gomory algorithm only deals with 1D bin-packing, we follow its philosophy of using column generation approach and decomposing our model into a master problem (cutting stock) and a sub-problem (pricing problem).

Our model (Eqs (16) to (22)) contains *k*! symmetric solutions and there are many binary variables, that makes problem extremely hard. To make this problem simpler, we can transform it to a cutting stock problem: instead of focusing on which kernel is put in a particular part of a batch, we look at possible *patterns* used to put in a batch. The question is then changed to focus on how many times a particular pattern is used:

$$\begin{array}{cc} \text{Minimize} & Z=\sum_{j}x_{j} \end{array}$$ (23)

$$\begin{array}{cc} \text{subject}\;\text{to} & \sum_{j}P_{ij}x_{j}\geq d_{i},\forall i \end{array}$$ (24)

$$\begin{array}{cc} & x_{j}\geq 0 \end{array}$$ (25)

In this model, *i* is the number of same kernels, *j* is the number of different types of patterns. *x*_*j*_ stands for number of *j*th pattern that has been used, *P*_*ij*_ stands for cutting pattern that *i*th kernel used in *j*th pattern, *d*_*i*_ stands for demands of kernel *i*.

It is natural to consider Simplex Algorithm as the solution [61]. However, there are 2^*i*^ − 1 patterns of the required *i* kernels [62]. Even if we had a way to generate all patterns, it is difficult to contain all variables into the algorithm. Thus for each iteration of in the Simplex Algorithm, we need to find the most negative column [60]. By defining a new sub-problem, we are able to find it.

$$\begin{array}{cc} \text{Minimize} & z_{sub}=1-\sum_{i}\pi_{i}P_{ij} \end{array}$$ (26)

$$\begin{array}{cc} \text{subject}\;\text{to} & \sum_{i}P_{ij}w_{i}\leq W \end{array}$$ (27)

$$\begin{array}{cc} \text{subject}\;\text{to} & \sum_{i}P_{ij}r_{i}\leq R \end{array}$$ (28)

$$\begin{array}{cc} \text{subject}\;\text{to} & \sum_{i}P_{ij}s_{i}\leq S \end{array}$$ (29)

$$\begin{array}{cc} & P_{ij}\in Z^{+} \end{array}$$ (30)

Here, *π*_*i*_ stands for the average demands of kernel *i* in this round of Simplex Algorithm. The sub-problem is a pricing problem as well as a three-dimensional knapsack problem, we can use dynamic algorithm similar to our algorithm in ***Algorithm of Kernel-optimization model*** and the complexity is *O*(*nWRS*). Hence, the Column Generation Algorithm for solving our pre-processing model can be seen in above Algorithm 2.

**Algorithm 2**: The Column Generation Algorithm

1: Initialize patterns

2: **repeat**

3:  Substitute patterns into master problem Eq (23), find *π*

4:  Solve sub-problem Eq (26), get new pattern

5:  Add new pattern to master prbolem

6: **until**

7: *z*_*sub*_ ≥ 0

## Experimental Evaluation

### Experimental setup and benchmark

We run all experiments in a server with a Intel(R) Xeon(R) CPU E5-2620 v3 @ 2.40GHz CPUs, 384 GB of DDR4 2133 MHz memory, equipped with two 400GB INTEL SSDSC2BX400G4 SSDs, two 4TB Western Digital Red disks, and eight NVidia GeForce GTX TITAN X (Pascal) graphics cards. The machine runs CentOS 6.6 and CUDA version 5.0 (MultiQx-GPU is only compatible with CUDA 5.0).

In the experiments, we compare the performance of our Two-stage Optimization Model with the solution provided by MultiQx-GPU and another baseline: **sequential execution** of kernels—this simulates the behavior of a typical resource schedulers where each application is treated as an independent process. In this setup, kernel parameters are set according to our previous work [20] to ensure best single-kernel performance.

As to the benchmark, since in-database analytics becomes popular [10–12], we uses both SQL queries and analytic queries from [17] and our previous work [63]. The detailed benchmarks are listed in Table 1. We picked different combinations of queries and measured the performance. Each experiment with same combination runs 400 times.

**Table 1:** Queries in benchmark.

Number	Queries
*Q*1	Hash join of two tables
*Q*2	Hash join of three tables
*Q*3	md5 verification
*Q*4	Matrix multiplication
*Q*5	Distance calculation of atomics

### Experimental results and discussions

Since we have proved in ***Algorithm of Kernel-optimization model*** that our algorithm will find the solutions with the largest number of active threads, discussions on experimental results will be focused on actual (total) running time of the workload. However, we want to first point out that, in all experimental runs, our solutions did reach the highest thread concurrency without exception. Moreover, if those combinations of kernels in which a feasible solution can be found in first batch, we will only apply Kernel-optimization model, otherwise, we will apply Batch-optimization model followed by Kernel-optimization model.

**Fig 7:**
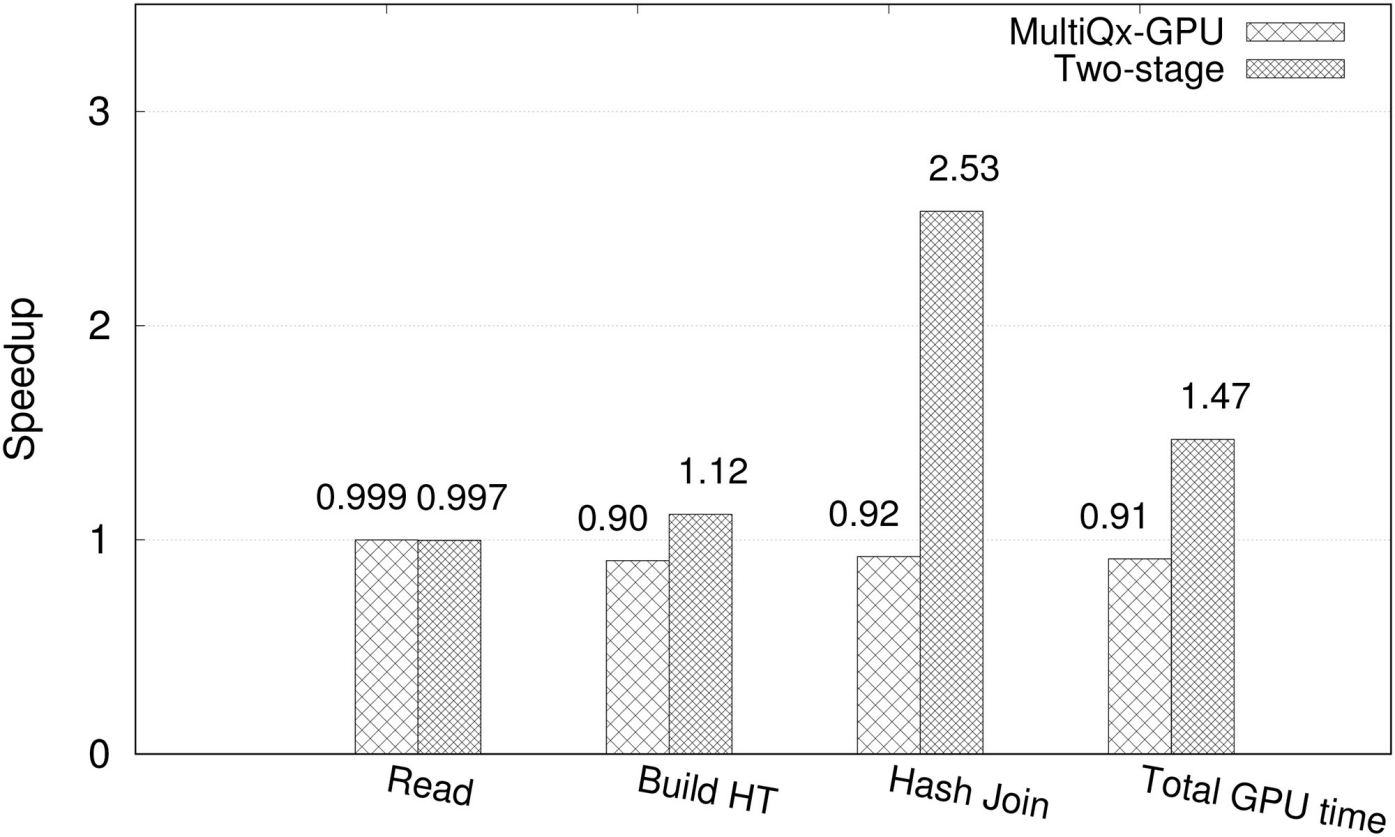
Speedup of two-table hash join GPU operations that MultiQx-GPU Optimization and Two-stage Model over sequential solution.

First, let us compare the performance of running Q1. As we mentioned in ***Introduction***, hash join is a complex operation involving several kernels (Fig 3). There are reading table (kernel I and IV), building hash table (kernel II and V), hash join (kernel III and VI). It can be naturally executed in parallel manners. As shown in Fig 7, we can see that Two-stage Model gains speedups of operation that take places on GPU against sequential execution while MultiQx-GPU has almost same performance with sequential execution. The average speedup of reading table, building hash table, and hash join against sequential execution of MultiQx-GPU and Two-stage Model are 0.999x and 0.997x, 0.90x and 1.12x, and 0.92x and 2.53x, respectively. The average speedup of all operations on GPU that MultiQx-GPU and Two-stage Algorithm achieve over sequential solution is 0.91x and 1.47x, respectively. It is not surprising to see that there is not much speedup of reading table since kernel I and kernel IV are still executed in sequential manner even though we use CUDA streams like in Fig 3.

**Fig 8:**
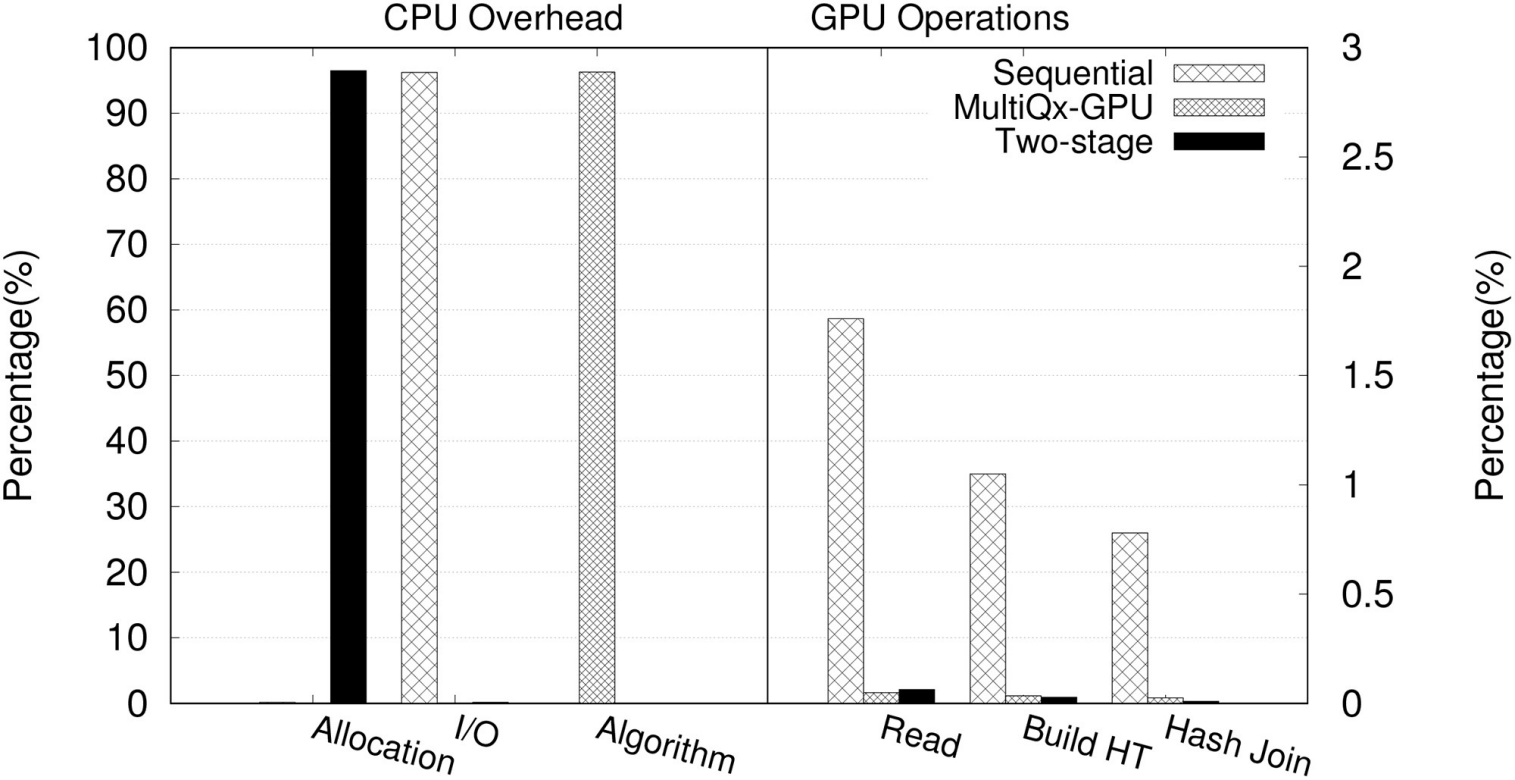
Percentage of execution time of two table hash join with sequential execution, MultiQx-GPU Optimization and Two-stage Model.

Similar to other GPU executions, the large overhead comes from CPU side. The execution breakdown time of three solution is shown in Fig 8. We can see that sequential solution has large overhead of I/O (transfer data between CPU and GPU); MultiQx-GPU transfers the overhead to initializing itself by creating a resource management environment on CPU, however, the overhead is a one-time cost, which means the ratio of its overhead can be reduced when executing multiple queries; as for Two-stage Model, its algorithm overhead is only 0.08 ms, but it costs long time to allocation memory on CPU, this is because to overlap data transfer between CPU and GPU, we need to pin memory on CPU side, which moves the overhead from I/O to memory allocation, note that, its overhead can be hidden in overlapping under a multi-query environment. Comparing the calculation on GPU side, the large overhead in Q1 is dominated and inevitable, thus the overall performance doesn’t have much difference.

However, the I/O overhead of sequential execution, algorithm overhead of MultiQx-GPU, and memory allocation overhead of Two-stage Model are almost same, creating a truly concurrent multi-kernel execution on GPU by Two-stage Model can improve the performance on GPU side.

**Fig 9:**
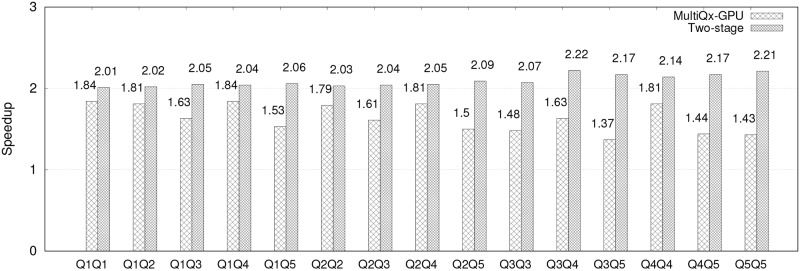
Speedup of two queries combinations that MultiQx-GPU Optimization and Two-stage Model over sequential solution.

Similar to [17], we compare the performance of MultiQx-GPU and our Two-stage model with sequential execution in multi-query workload that has each combinations of two queries. Such results are presented in Fig 9. We can see that performance of both models have improved in a multi-query environment. For example, when executing Q1 and Q2 concurrently, MultiQx-GPU has a speedup of 1.81x and Two-stage model has a speedup of 2.02x. The results support our theory that CPU-side overhead of both MultiQx-GPU and Two-stage model can be hidden under multi-query environment. As compared to the sequential solution, the speedup of Two-stage model is at least 2.01x, and the speedup of MultiQx-GPU is at least 1.37x. The reason that Two-stage model has better performance than MultiQx-GPU is because our model not only enables overlaps in resource allocation on CPU but also overlaps in kernel executions on GPU.

**Fig 10:**
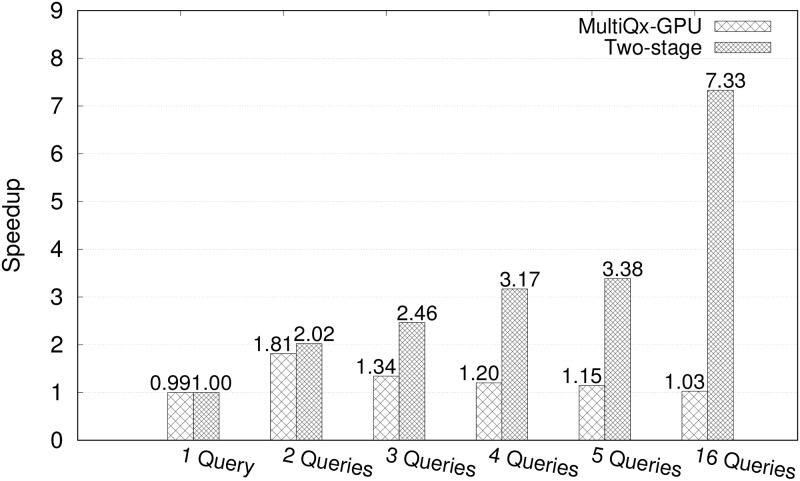
Speedup of different number of queries that MultiQx-GPU Optimization and Two-stage Model over sequential solution.

By observing, we can see the average speedup of Two-stage model under two-query workload is around 2.0x, will the performance further improve with more queries? We test the speedups of MultiQx-GPU Optimization and Two-stage Model over sequential solution under workloads with different number of queries. The workload is generated by repeatedly picking queries from Q1 to Q5 in order based on the number of queries in a workload we need (Like, for a seven-query workload, we’ll pick Q1Q2Q3Q4Q5Q1Q2). The results are presented in Fig 10. We can see that the speedup of MultiQx-GPU over sequential execution reaches maximum value (1.81x) under two-query workload then goes down to 1.15x withnin five-query workload. While the speedup of our Two-stage model over sequential execution increases with the number of queries increase, from 1.00x under one-query workload to 3.38x within five-query workload. The difference between two approaches is our method enables both CPU-side (memory allocation, I/O) overhead overlapping and GPU-side kernel execution overlapping, thus the increase of Two-stage model is nearly linear while MultiQx-GPU decreased after two-query workload. With the number of queries increased, the advantage gained from our model is more obvious. We can see from a 16-query workload, our model achieves 7.33x speedups while MultiQx-GPU has 1.03x speedups.

As a special note, the running time reported above includes the time for solving the optimization model. The computational overhead of such solutions, as shown in our analysis (***Algorithm of Kernel-optimization model***), is minimum. In particular, the time to solve the optimization in all our experiments range from 0.082 ms to 1.487 ms and the average time is 0.571ms.

## Conclusions

With very high parallel computing capacity, GPUs have become an integrated part of today’s HPC systems and found applications in many scientific and computing domains. Management of large-scale scientific data has seen in-memory databases and push-based query engine design as the main approach in dealing with the I/O bottleneck. By feeding shared data streams to multiple concurrent queries, such systems removed the bottleneck from I/O to computation, making GPUs a suitable platform for running the query engine. A key challenge in the implementation of such systems is to support concurrent tasks. Task parallelism feature (i.e., the CUDA stream) provided by CUDA can be leveraged to meet such challenges. The objective of this study is to allocate resources to concurrent CUDA kernels by configuring their runtime parameters for the purpose of maximizing system performance. We develop an integer programming model to describe such a problem and design algorithm for solving the optimization with proved correctness and high efficiency.
